# The Use of Pectoralis Major Musculocutaneus and Deltopectoral Flaps in Oromandibular Defects Reconstruction

**DOI:** 10.29252/wjps.8.3.401

**Published:** 2019-09

**Authors:** Mahammad M Davudov, Chingiz Rahimov, Hamidreza Fathi, Zoheyr Mirzajani, Mirvari Aliyeva

**Affiliations:** 1 Department of Oral and Maxillofacial Surgery, Azerbaijan Medical University, Baku, Azerbaijan;; 2 Department of Aesthetic, Plastic and Reconstructive Surgery, Tehran University of Medical Sciences, Tehran, Iran

**Keywords:** Pectoral major musculocutaneus, Flap, Deltopectoral, Osteoradionecrosis, Mandible, Reconstruction

## Abstract

The main complication in patients with combined treatment of head, neck, mandibular and maxillary tumors is osteoradionecrosis, which appears after radiation therapy. Radiation therapy is widely used to treat cancer, but growing concern is related to the risk of osteoradionecrosis after treatment. This can occur after radiation therapy. Below, we would like to describe the treatment of osteoradionecrosis, which appeared 5 years after radiation therapy in a 54-year-old male patient. In 2012, a patient in Turkey was diagnosed with adenocystic carcinoma of the tongue base, and surgery was performed to remove the tumor after the patient underwent a course of radiotherapy. In 2016, the patient underwent again a surgery for tumor recurrence. In December 2017, the patient was admitted to our clinic with osteoradionecrosis. We performed segmental resection of the mandible, type I right-sided modified neck dissection, reconstruction of the mandible with a titanium plate and a pectoralis major muscle skin flap. The technique described in this case is the insertion of a well-vascularized tissue into the pre-irradiated and necrotic hypovascular region of the mandible with a skin-muscle flap of the pectoralis major muscle wrapped around the plate for reconstruction. As a result, a pectoralis major flap coverred the mouth floor on internal side and the outside skin defect was covered with a deltopectoral one. The viability of the skin-muscle flap of the pectoralis major muscle was assessed using clinical monitoring, checking the flap every four hours for the first 3 days. This study describes a successful outcome.

## INTRODUCTION

One of the main points of discussion around the tissues radiation damage is the obliteration of blood vessels, which leads to tissue hypoxia, as a result to hypovascularization and hypocellularity, which is characterized by fibrosis and bone necrosis in the treatment area. Osteoradionecrosis (ORN) is defined as the area of damaged, incompetent bone in the radiation area, which can lead to fractures. In most cases, these fractures do not heal with the use of conservative treatment for 4 months or more.^1^^-^^3^

If the treatment involves only the use of a titanium plate, then sooner or later it will inevitably lead to a fracture of the mandible. In our clinic, in such cases, the skin-muscular flap of the pectoralis major muscle is the main choice for reconstruction after mandible resection. However, the condition of the blood vessels at the donor site and previous surgical interventions may limit the use of the free flap.^1^^-^^3^ The use of skin-muscle flaps of the pectoralis major and deltopectoral flaps is more suitable for the reconstruction of intraoral and extraoral defects of soft tissues.^4^^-^^6^ These patches have no limitations providing the necessary combination of bone and soft tissue.^7^^-^^9^

The functional results of the surgical procedure are dictated by the technique used for the reconstruction of the lower jaw by a titanium plate, bone and free flap.^10^^-^^12^ We present a technique for modeling the lower jaw by a skin-muscle flap of the pectoralis major muscle and the deltopectoral flap. In the present case, the treatment conditions of our patient for a free unvascular bone graft are not so ideal due to poor blood supply after radiotherapy. The patient was operated once and received adjuvant radiotherapy. Taking into account the fact that was radionecrosis area and vessels were fibrotic, the reconstructive plate and pectoral major flap were chosen as alternatives to microvascular tissue transfer. 

These problems prompted us to look for new ways to create a recipient bed with an adequate choroid for obtaining a bone graft in the form of a skin-muscle flap of the pectoralis major muscle with the success of primary healing and subsequent functional shaping of the bone. The technique described in this case is the insertion of a well-vascularized tissue into the pre-irradiated and necrotic hypovascular region of the mandible with a skin-muscle flap of the pectoralis major muscle wrapped around the plate for reconstruction. Thus, the place of the recipient of a non-oriented well vascularized soft tissue is formed, which can cover the intraoral defect with secondary epithelialization of the pectoral muscle and the external defect with the skin-muscle flap. Extensive and complex intraoral and extraoral defects of soft tissues represent another possible area of application of the skin-muscle flap of the pectoralis major muscle.^13-15^ The use of this technique makes it possible to better restore the height of the lower jaw.

## CASE REPORT

**Fig. 1:**
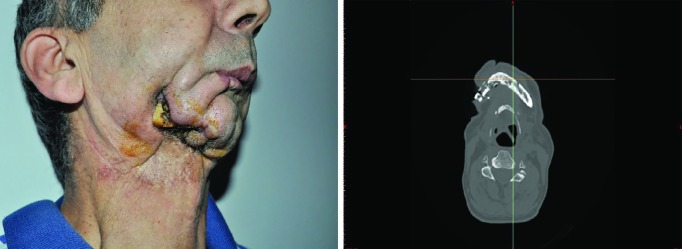
Clinical photo and CT scan of a patient with osteoradionecrosis

We would like to describe the treatment of osteoradionecrosis, which appeared 5 years after radiation therapy in a 54-year-old male patient (Figure 1).

**Fig. 2:**
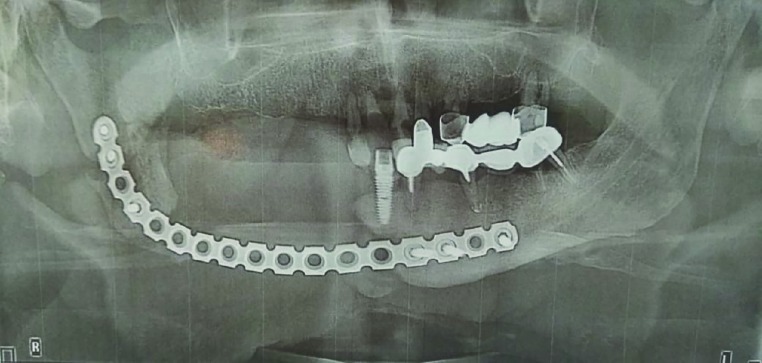
OPG of patient after surgery (segmental resection of mandible and insert the defect titanium reconstructive plate).

We have performed a resection of the mandible segment and right-sided neck dissection, together with use of pectoralis major muscle vascularized muscle-skin flap for reconstruction. In this case, the method of pretreatment was applied, simultaneously with the reconstruction of the lower jaw. The side edge of the right side of the lower jaw was exposed and broken, if necessary for bone resection, as well as for insertion and fixation of the flap for reconstruction. A minimum of 3 cm was required for fixing a 3 mm titanium plate attached to the sides of the lower jaw (Figure 2). 

Affected soft tissue was removed to form safe fields. Next step was the segmental resection of the lower jaw. It is advisable to fix the plate on each side of the lower jaw with the help of three screw holes (Figure 2). The flap was separated from the donor site and shaped according to the recipient area. The collection time of the flap ranged from 40 to 100 minutes. A simulated pectoral major muscle-skin flap was inserted between the segments of the lower jaw. During the design of the flap, a leather protrusion was formed above the pectoral muscle. The cutaneous was protruded on the pectoral muscle splits, and the incision was continued along the anterior axillary fold. 

**Fig. 3:**
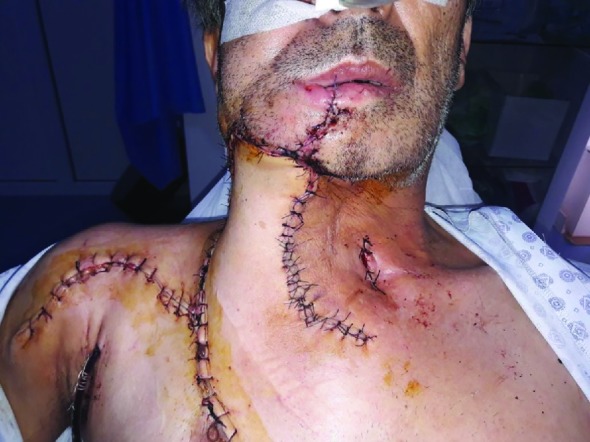
View of patient, 1 week after surgery

The pectoralis major muscle was risen along the lateral edge of the pectoralis major muscle. The flap was usually carried out in the neck surface superficially to the clavicle through a wide subcutaneous tunnel. It should be checked that the leg did not twist during the operation. Sometimes, the tunnel can be narrow, but it can be expanded by making incisions through the medial and distal ends of the periosteum, which makes it easy to pass. As a result, pectoral major muscle-skin flap coverd the inside of the floor of the mouth, and the outside of the skin defect was covered with delto-pectoral flap (Figure 3). 

**Fig. 4:**
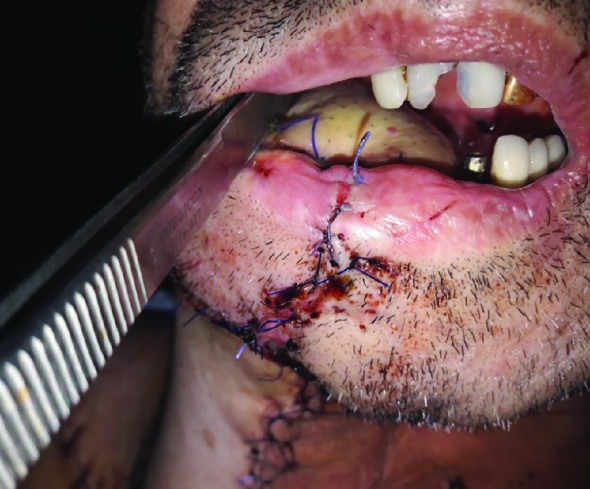
PMMF insert to oral site of oromandibular defect

**Fig. 5:**
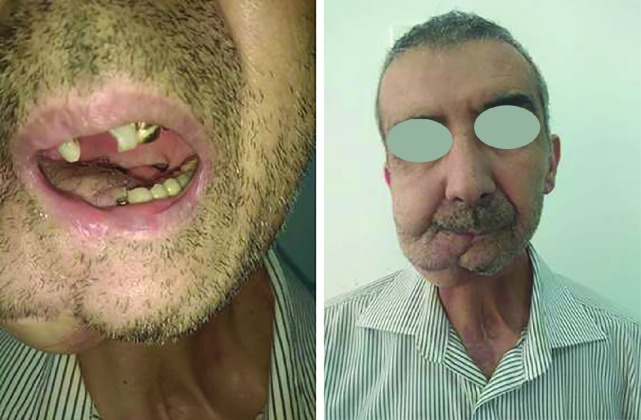
Intraoral (A) and extra oral (B) view of patient after remodeling pedicles of pectoral major and deltopectoral flaps

In the postoperative period, the correct occlusion of the teeth was confirmed by the assessment of the oral cavity and computed tomography. The viability of the skin-muscle flap of the pectoralis major muscle was assessed using clinical monitoring, checking the flap every four hours for the first 3 days (Figure 4). After 6 months, a flap pedicle was remodeled (Figure 5A and B)

## DISCUSSION

All surgical procedures performed during the reconstruction of the mandible are an important link to achieve ideal functional results. The presence of at least three screws fixing the plate for each segment of the lower jaw avoids the dislocation of the residual mandible and guarantees the maintenance of the jaw profile and the correct occlusion of the teeth. The main role in the reconstruction of the lower jaw was played by the anatomical features of the localization of the defect. The muscular-dermal flap of the pectoralis major muscle has been used in a variety of reconstructive procedures of head and neck, which may include covering mucosal defects and skin defects.^4^^-^^6^

It can be used for two epithelial surfaces at one stage of reconstruction without changing the position of the patient during the surgical procedure. The flap of the pectoralis major muscle can be used as a separate muscle or muscle-skin flap. This flap reduces the impact of ischemia in the operating area and allows you to gain more time for the formation of microanastomoses. In our technique, we propose to separate the skin-muscle flap of the pectoralis major muscle and further work on its shaping. We were convinced that the risk of damage when using the vascular pedicle during the simulation of the titanium plate was higher than when it was still attached to the donor by the vessels.^7^^-^^10^

When using this method, the time of ischemia never exceeded 90 min, and intra-operative monitoring of the flap confirmed the viability of the flap during surgery. Reconstruction of the lower jaw using the skin-muscular flap of the pectoralis major muscle may contribute to optimal functional results.^11^^-^^15^ Although every step in flap shaping is important, this is a versatile, reliable and economical method using a flap with an excellent choroid, a wide arch of rotation, a large flap size and ease of material collection. At the same time, the risk of complications is minimal.
